# Local and Systemic Injections of Human Cord Blood Myeloid-Derived Suppressor Cells to Prevent Graft Rejection in Corneal Transplantation

**DOI:** 10.3390/biomedicines10123223

**Published:** 2022-12-12

**Authors:** Jae-young Lee, Hyun-Jung Sohn, Chang-Hyun Kim, Tai-Gyu Kim, Hyun Soo Lee

**Affiliations:** 1 Department of Ophthalmology, Eunpyeong St. Mary’s Hospital, College of Medicine, The Catholic University of Korea, Seoul 03312, Republic of Korea; 2 ViGenCell Inc., Seoul 06591, Republic of Korea; 3 Catholic Hematopoietic Stem Cell Bank, College of Medicine, The Catholic University of Korea, Seoul 06591, Republic of Korea; 4 Department of Molecular Medicine, The Scripps Research Institute, 10550 North Torrey Pines Rd., La Jolla, CA 92037, USA

**Keywords:** Myeloid-derived suppressor cells (MDSCs), corneal transplantation, graft rejection, T cells, macrophages

## Abstract

Myeloid-derived suppressor cells (MDSCs) are therapeutic agents to prevent graft rejection in organ transplants by modulating inflammation. Herein, the immunosuppressive effect of human cord blood MDSCs on corneal allograft models was confirmed. CB-MDSCs were locally (subconjuctival, 5 × 10^5^) or systemically (intravenous, 1 × 10^6^) injected twice on days 0 and 7. A corneal transplantation model was established using C57BL/6 and BALB/c mice, and corneal graft opacity was measured to evaluate graft rejection up to 6 weeks. Results showed that graft survival in the MDSCs groups increased compared to vehicle groups after 42 days. Systemic and local MDSC administration inhibited the maturation (MHC-II^hi^ CD11c+) of dendritic cells (DCs) and the differentiation of interferon γ+ CD4+ Th1 in draining lymph nodes (LNs). However, vehicle groups increased the infiltration of CD3+ T cells and F4/80+ macrophages and produced prominent neovascular and lymphatic vessels into the graft site with increased mRNA expression of VEGF-A/C and VEGFR-1/R-3. Local MDSCs administration showed prominent anti-angiogenic/anti-lymphangiogenic effects even at lower MDSCs doses. Thus, CB-MDSCs could relatively suppress the infiltration of pathological T cells/macrophages into the corneas and the migration of mature DCs into draining LNs Therefore, ocular and systemic MDSCs administration showed therapeutic potential for preventing corneal allograft rejection.

## 1. Introduction

Penetrating keratoplasty (PK) is the most frequently performed transplantation internationally [1]. In this procedure, the diseased corneal opacity is replaced with a healthy donor cornea to make daily life easier for people with vision loss. However, corneal graft rejection is a major cause of graft failure, and graft rejection occurs in 30–40% of general corneal transplantation cases [2,3,4]. Rejection rates are significantly higher in high-risk cases, thereby increasing vascularized beds within the recipient cornea [5,6]. The cornea is an immune-privileged state that can be compromised by inflammation, injection, or neovascularization after corneal transplantation [7]. Therefore, immunosuppressants such as steroids, cyclosporine, and tacrolimus are clinically used to control acute rejection after transplantation. However, the long-term use of steroids and immunosuppressants is associated with serious problems such as diabetes, steroid-induced glaucoma, secondary infection, and cataracts [8,9]. Therefore, the development of additional therapeutic agents to minimize the use of immunosuppressants and maintain long-term immune tolerance is of clinical importance.

In allogeneic corneal transplantation immunity, CD4+ helper 1 cells and F4/80+ macrophages mainly contribute to transplant rejection. CD11c+ dendritic cells (DCs) as potent antigen-presenting cells (APCs) that recognize alloantigens activate T cell-mediated adaptive immune cells for immune-mediated graft rejection [10]. In addition, macrophages enable the migration of immune cells between the eyes and draining lymph nodes by inducing angiogenesis and lymphangiogenesis, which are initiated by ocular inflammation postoperatively [11,12]. In the adaptive immune system, activated CD4+ T cells produce inflammatory cytokines (such as IFN-r and IL-17), amplify the influx of innate immune cells, and lead to corneal allograft rejection [13]. Apoptotic cells in corneal transplant rejection are directly caused by infiltrating CD4+ and CD8+ T cells, which destroy tissues in a cytokine-mediated manner [14]. In addition, an increased MHC class II expression of APCs is an important factor that influences the rate of Th1-mediated graft rejection through the proliferation of alloantigen-specific T cells [15,16]. A strong immune response that disrupts corneal immune privilege in this way is considered a type 4 hypersensitivity reaction that may exacerbate graft rejection [3]. Therefore, APCs maturation and CD4+ cell proliferation in corneal transplantation should be suppressed to increase graft survival outcomes.

Myeloid-derived suppressor cells (MDSCs) are heterogeneous populations of immature myeloid cells, including myeloid progenitors, to control excessive inflammation under pathological conditions such as chronic inflammation and cancer-mediated inflammation [17,18,19]. MDSCs of various origins have shown promising results as therapeutic immunomodulators [17,18,19]. Studies have elucidated the suppressive mechanism of the activation and proliferation of T cells through inducible nitric oxide synthase (iNOS) and arginase 1 (Arg-1) expression of MDSCs. MDSCs also induce regulatory T cells (Tregs) that regulate or suppress T cells in the immune system [20,21]. Park et al. [22] reported that human-derived cord blood (CB)-MDSCs present a potent immunosuppressive function on T cell-mediated chronic inflammation in a mouse graft-versus-host disease (GvHD) model through immunosuppressive molecules, including iNOS, Arg-1, and indoleamine 2,3-dioxygenase (IDO). MDSCs have attracted attention as important suppressive regulators of non-neoplastic inflammation, such as inflammatory bowel diseases (IBD), acute kidney injury, and autoimmune uveitis mouse models [23,24,25]. Therefore, human-derived CB-MDSCs on corneal transplantation might show potential for therapeutic applications and preventive methods to increase graft survival.

In this study, we investigated the therapeutic efficacy of locally or systemically administered CB-MDSCs to suppress corneal allograft rejection by evaluating immunological responses and the compromised immune privilege after corneal transplantation in a murine model.

## 2. Materials and Methods

### 2.1. Animals

BALB/c and C57BL/6 female mice (6–8 weeks old; Koatech Bio. Inc., Pyeongtaek, Korea) were housed in a specific pathogen-free environment for 1 week. All experiments were conducted in accordance with the guidelines of the Catholic Institutional Animals and the ARVO Statement for the Use of Animals in Ophthalmic and Vision Research (IACUC Approval no. EPS-MH-2020-1701-FA). Anesthesia was intraperitoneally induced by 120 mg/kg ketamine and 20 mg/kg xylazine.

### 2.2. Murine Model of Corneal Transplantation

Corneal Transplantation (PK) was performed on BALB/c mice as recipients of corneal allografts as previously described [26]. Briefly, the central cornea of C57BL/6 (donor) mice was excised using a 2 mm biopsy punch, and the central cornea of BALB/c (recipient) mice was excised for transplantation with a 1.5 mm biopsy punch. The donor corneal graft was then sutured to the center of the recipient cornea by using six interrupted 11-0 nylon sutures (Sharp Point, Reading, PA, USA). The eyelids of all recipient mice were sutured by tarsorrhaphy 3 days after surgery; all corneal sutures were removed 7 days after transplantation. Graft rejection was evaluated by clinical scoring under a microscope. Grading was based on a scale of 0–4 in terms of the presence and extent of opacity, as previously described [26]. Graft rejection was confirmed as two successive scores of ≥3, indicating the obscured iris shapes. Graft survival graphs were modeled using Kaplan–Meier survival curves.

### 2.3. MDSCs Administration

Human cord blood (CB)-MDSCs were provided by the ViGenCell Inc., (Seoul, Republic of Korea), and the Catholic Hematopoietic Stem Cell Bank, an affiliation of the College of Medicine, The Catholic University of Korea, which was approved by the Institutional Review Board of the Catholic University of Korea, College of Medicine (Permit No. MC17TNSI0002). hCB-MDSCs were generated ex vivo as described previously [23,27]. Briefly, neonatal umbilical cord blood was collected from the umbilical veins after maternal informed consent. After CD34+ cells were isolated with a magnetic cell-sorting system (anti-human CD34 antibodies (isotype: mouse IgG1), cat# 130-046-702, Miltenyi Biotec, Bergisch Gladbach, Germany) were cultured with Iscove’s Modified Dulbecco’s Medium medium, combined with 10% heat-inactivated fetal bovine serum (Gibco, Thermo Fisher Scientific, Waltham, MA, USA), 2 mM L-glutamine (Lonza), recombinant human granulocyte-macrophage colony-stimulating hormone (100 ng/mL), and recombinant human Stem Cell Factor (50 ng/mL) (Peprotech, Rocky Hill, NJ, USA). After 6 weeks of culture, about 90% of hCB-MDSCs expressed CD11b+CD33+ CD14^+^ HLA-DR^low/−^ through flow cytometry [23,27]. Human CB-MDSCs were injected via 5 × 10^5^ subconjunctival (subconjunctival; scj) or 1 × 10^6^ via tail vein (intravenous; iv) in the corneal transplanted mice. The control groups received an equal volume of vehicle (PBS) via local (scj) or systemic (iv) injection. Each injection was administered via a subconjunctival or intravenous route on day 0 (1st injection) and day 7 (2nd injection) post-transplantation, respectively.

### 2.4. Immunofluorescent Staining

The mice were dissected on week 6 for whole eye tissue section experiments immediately after euthanasia. The whole eye tissues were fixed in 4% paraformaldehyde (Cat# BPP9004, Tech and Innovation, Gangwon-do, Korea), precipitated on sucrose (Cat# S9378, Sigma-Aldrich, Hamburg, Germany), and embedded using O.C.T compound (Cat# 4583, Sakura Finetek, CA, USA) in a cryo-mold. The embedded eye tissues were sectioned on a cryo-microtome at 8 μm thickness and attached to glass slides. The cryo-sectioned corneas were incubated in a SuperBlock blocking solution (ThermoFisher, Rockford, IL, USA) at 20–25 °C for 30 min to block nonspecific staining. The corneas were immunostained with Alexa Fluor 594-anti-mouse CD3 antibody (1:200, host:mouse, cat# 100240, BioLegend, San Diego, CA, USA), Alexa Fluor 488-anti-mouse F4/80 antibody (1:200, host:mouse, cat# 123120, BioLegend, San Diego, CA, USA) overnight at 4 °C and mounted using a Vector Shield mounting medium containing DAPI (cat# H-1200, Vector Laboratories, CA, USA). For the TUNEL assay, the cryo-sections of the corneas were blocked in the SuperBlock solution (ThermoFisher, Rockford, IL, USA) at 20–25 °C for 30 min and incubated for permeabilization with PBST (0.1 Triton X in PBS) at 37 °C for 20 min. The permeabilized corneas were stained via a terminal deoxynucleotidyl transferase dUTP nick end labeling (TUNEL) staining kit (cat# 11684795910, Roche, Basel, Switzerland) in accordance with the manufacturer’s recommendations. TUNEL-stained tissue was also mounted using a Vector Shield mounting medium with DAPI. Frozen sections of corneas were examined under a fluorescence microscope (Axiovert 200, Zeiss, Germany) in a blinded fashion at 20× magnification. Corneal photographs were quantified as the average of three photographs randomly selected for each animal, and each group included four animals in a masked fashion. 

### 2.5. Corneal Whole Mount Staining by Immunofluorescence

Six weeks after transplantation, freshly obtained corneas were fixed in acetone at 20–25 °C for 15 min. The whole cornea was stained overnight at 4 °C with primary antibodies, FITC-conjugated anti-mouse PECAM-1 antibody (1:100, host:rat, cat# sc-18916, Santa Cruz, CA, USA), and Alexa Fluor 594-conjugated anti-mouse LYVE-1 antibody (1:100, host:rat, cat# FAB2125T, R&D Systems, Minneapolis, MN, USA). All cornea mounts were trimmed using a blade to flatten the cornea and mounted on slides by using the Vector Shield mounting medium containing DAPI. Each whole cornea was examined with a fluorescence microscope at 5× magnification (Axiovert 200, Zeiss, Germany). The area within the grafted cornea covered by blood vessels (CD31) and lymphatic vessels (LYVE-1) was calculated using ImageJ 1.52v (National Institute of Health, Bethesda, MD, USA). Corneal CD31^low^ LYVE-1^hi^ vasculature represented the lymphatic vessels, and CD31^hi^ LYVE-1^low^ vasculature represented the blood vessels. Neovascular perfusion (%) and lymphatic neovascularization (%) areas were calculated through normalization of the total corneal area by combining the sub-areas in the photographs of each individual mouse under blinded random selection [23,24].

### 2.6. Real-Time Polymerase Chain Reaction (PCR)

PK mouse corneas were harvested 6 weeks after surgery, and three or four corneas were randomly collected within each group. Total RNA was isolated from the transplanted corneas by Trizol (Invitrogen, Carlsbad, CA, USA) and RNeasy Mini (Qiagen, Hilden, Germany). cDNA was then reverse transcribed from total RNA by using SuperScript III™ Reverse Transcriptase (Invitrogen, Carlsbad, CA, USA). Real-time PCR was performed with Taqman PCR Mastermix and FAM dye-labeled predesigned primers (VEGF-A: Mm00437306_m1, VEGF-C: Mm00437310_m1, VEGF-R2: Mm01222421_m1, VEGF-R3: Mm01292604_m1, glyceraldehyde 3-phosphate dehydrogenase (GAPDH): Mm99999915_g1, ThermoFisher, Rockford, IL, USA). GAPDH was used as an internal control for each reaction. Gene expression levels were analyzed via the comparative threshold cycle method using the analysis software (Quantity One 1-D analysis, Bio-Rad, Hercules, CA, USA), and relative expression levels for each sample were expressed as fold change compared with the untreated naive mice.

### 2.7. Flow Cytometry Assay

Draining LNs were isolated from graft recipients on postoperative day 42, and a single-cell suspension was passed via a 70 μm cell strainer (Corning, CA, USA). Viable single cells were subjected to plate counting as 5 × 10^5^ cells/well in 96-well plates on RPMI media (Welgene Inc., Gyeongsan-si, Republic of Korea) with 1% fetal bovine serum (FBS; Gibco BRL, Karlsruhe, Germany) for 48 h. The cultured single cells were harvested and immunostained with the following antibodies: PE-anti-mouse CD11c antibody (1:100, host:hamster, cat# 117307, BioLegend, San Diego, CA, USA), Alexa Fluor 647-anti-mouse I-Ad antibody (1:100, host:mouse, cat# 115010, BioLegend, San Diego, CA, USA), Alexa Fluor 488-anti-mouse CD4 antibody (1:100, host:mouse, cat# 100423, BioLegend, San Diego, CA, USA), and PE-anti-mouse IFN-gamma antibody (1:100, host:rat, cat# 12-7311-82, Invitrogen, Carlsbad, CA, USA). All antibodies were stained appropriately with the matched isotype controls. The stained cells were analyzed with FACS Melody (BD Biosciences, Franklin Lakes, NJ, USA) and FlowJo software X 10.5.3 (FlowJo LLC, Ashland, OR, USA).

### 2.8. Statistical Analysis

Data normality was performed with the D’Agostino–Pearson test. Student’s t-test or one-way ANOVA with post hoc paired Tukey’s test was used to calculate the significance between groups. Kaplan–Meier analysis with log-rank test was conducted to evaluate graft survival. Data were expressed as the mean  ± standard error of the mean (SEM) and considered statistically significant at *p* < 0.05. Data were statistically analyzed using Prism version 5.0 (GraphPad, San Diego, CA, USA).

## 3. Results

### 3.1. Local and Systemic MDSCs Administration Enhanced the Corneal Graft Survival after Corneal Transplantation

**Figure 1:**
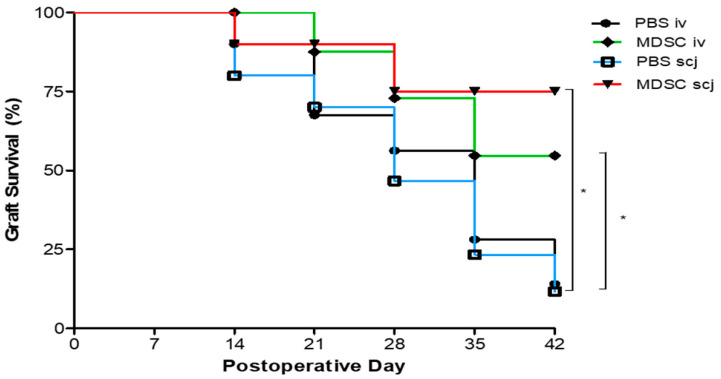
Comparison of corneal allograft survival according to the local and systemic administration of MDSCs. BALB/c (recipient) corneas were engrafted orthotopically onto C57BL/6 (donor) corneas by using PBSiv (*n* = 9), MDSCiv (*n* = 9), PBSscj (*n* = 11), and MDSCscj (*n* = 11). Locally and systemically injected MDSCs (scj, iv) groups displayed significantly better corneal allograft survival than PBS-injected (scj, iv) groups (* *p* < 0.05) for 42 days. Kaplan–Meier survival curves were obtained via the log-rank test for the statistical comparison of four groups.

The corneal allograft survival was evaluated by administering MDSCs locally or systemically. MDSCs suspended in PBS were administered via local (scj) or systemic (iv) injection, and the PBS (scj, iv, respectively) control group was injected with the same volume of the vehicle (PBS) on 0 days and 7 days. MDSC suspensions were administered locally (subconjunctival, 5 × 10^5^) or systemically (intravenous, 1 × 10^6^) on days 0 and 7 postoperatively in each group. Postoperatively, the grafted cornea of each recipient mouse was examined and clinically scored in a blinded manner every week. The objects added to each group were PBSiv (*n* = 9), MDSCiv (*n* = 9), PBSscj (*n* = 11), and MDSCscj (*n* = 11) that, were composed of screening individuals without infection, hemorrhage, and synechiae in all mice. The MDSC-injected groups had significantly better graft survival than the PBS-injected group (*p* < 0.05) at 6 weeks. The PBSiv and PBSscj groups showed less than 50% graft survival during the experiment, and the median survival time (MST) was 28.0 days. However, both local and systemic MDSCs-injected mice showed over 50% survival 6 weeks after surgery, and MST was undefined (Figure 1).

### 3.2. Local and Systemic MDSCs Suppressed DC Maturation and IFNγ-Expressing Effector CD4+ T Cell Generation in Draining LNs in the Corneal Transplantation Model

**Figure 2:**
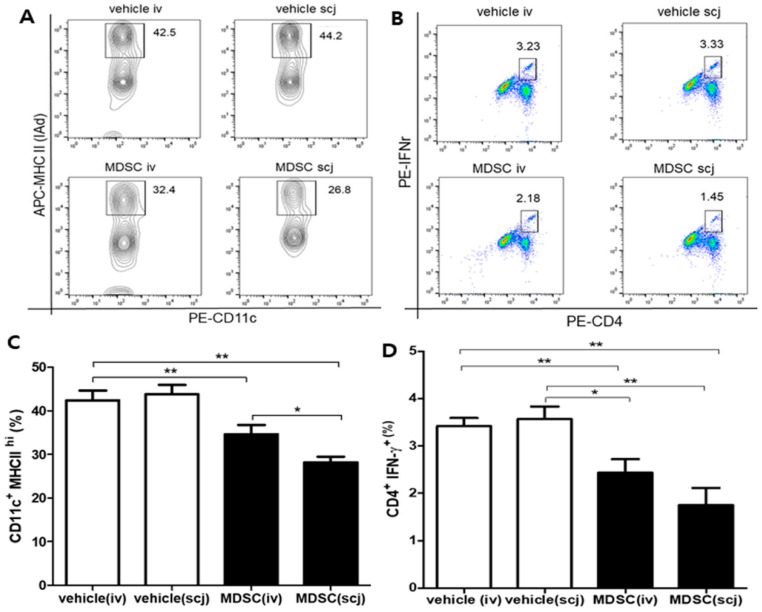
Populations of major histocompatibility complex (MHC) II^hi^ CD11c+ cells and differentiation of interferon (IFN)-γ-expressing effector T cells in draining lymph nodes after corneal transplantation. Populations of MHC II^hi^ CD11c+ dendritic cells (DC) and differentiation of interferon (IFN)-γ-expressing effector T cells in draining lymph nodes (LNs). (**A**) Representative flow cytometry plot showing MHC II^hi^ CD11c+ cells after CD11c+ gating. (**B**) Representative flow cytometry plot presenting the differentiation of IFNγ+ CD4+ cells. (**C**) Quantitative analysis of MHC II^hi^ CD11c+ cell populations (*n* = 3). Comparison between MDSCs (MDSC iv, MDSC scj) and vehicle (PBSiv, PBSscj) showed that the MDSCs groups presented a decreased maturation of MHC II^hi^ CD11c+ cells, whereas the vehicle groups did not. The immunomodulating effect of MDSCs was significantly better in the local (scj) injection group than in the systemic (iv) injection group. (**D**) Quantitative analysis graph of IFNγ+ CD4+ cell differentiation (*n* = 3). MDSCs groups (MDSC iv, MDSC scj) led to inhibit differentiation of IFNγ+ CD4+ cells when compared with vehicle groups (PBSiv, PBSscj). Representative flow cytometric data from three independent trials with pooled cells from three mice per group. * *p* < 0.05, ** *p* < 0.01.

The increased expression of MHC class II on DCs promotes T cell-mediated immune responses, which contributed to the dominant proliferation of IFN-γ+ CD4+ Th1 cells during graft rejection. Furthermore, IFN-γ+ CD4+ T cells are important in graft rejection by directly participating in graft tissue injury. Accordingly, flow cytometry was utilized to analyze CD11c+MHC-II^hi^ cells in the cornea, including the conjunctiva, and IFNγ+CD4+ T cell populations in draining LNs. The MHC-II^hi^ CD11c + cell population decreased significantly in the MDSCs groups (34.60 ± 1.22% for MDSCiv; 28.17 ± 0.75% for MDSCscj) compared with that in the vehicle groups (42.43 ± 1.27% for PBSiv; 43.80 ± 1.23% for PBSscj) (Figure 2A,C, *p* < 0.01). In addition, IFN-γ+ CD4+ T cell population significantly decreased in the MDSCs groups (2.48 ± 0.16% for MDSCiv; 1.75 ± 0.21% for MDSCscj) compared with the vehicle groups (3.42 ± 0.10% for PBSiv; 3.57 ± 0.15% for PBSscj; Figure 2B,D, *p* < 0.01). These data suggested that local and systemic administration of MDSCs can modulate the maturation of the MHC-II^hi^ CD11c+ cells and the proliferation of IFNγ-expressing effector Th1 cells dominant in corneal allograft rejection.

### 3.3. MDSCs Reduced Recruiting Macrophages and CD3+ T Cells in Grafted Corneas

**Figure 3:**
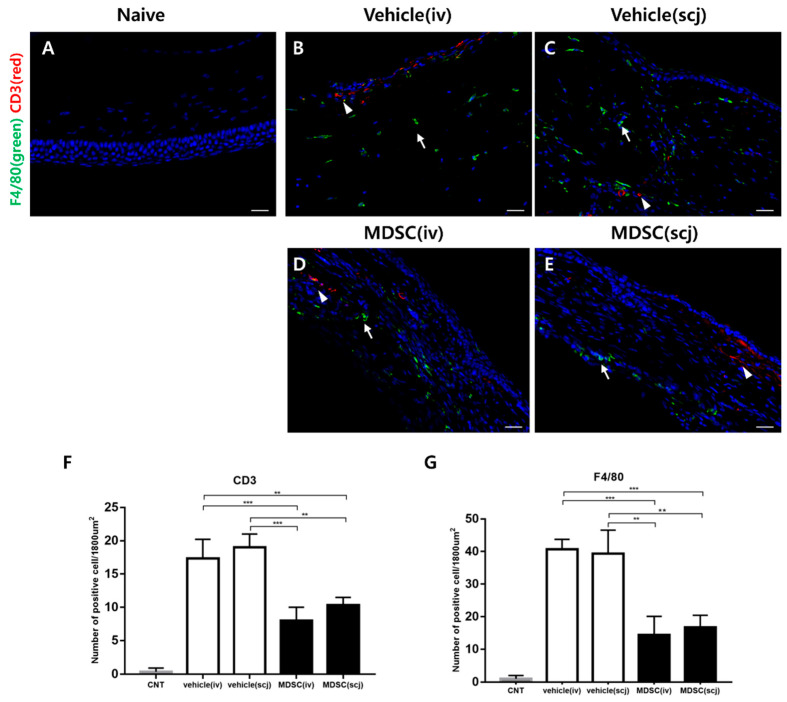
Immunohistochemical (IHC) staining of corneal allografts to evaluate infiltrating immune cells. (**A**) Representative IHC staining showed the naive group (**A**) and infiltration of CD3+ T cells (green; white arrow) and F4/80+ macrophages (red; white arrowhead) in the grafted corneas (**B**–**E**) on week 6 (20× magnification, scale bar = 50 µm). (**F**) Quantitative analysis of CD3+ T cell infiltration (*n* = 3). (**G**) Quantitative analysis of F4/80+ macrophages infiltration (*n* = 3). In the MDSCs groups, the infiltration of CD3+ T cells and F4/80+ macrophages in the grafted corneas was significantly suppressed regardless of the delivery site. Three to four different sections from three independent mice were randomly selected for counting blinded samples, and the average was calculated. Data were presented as average ± SEM. ** *p* < 0.01, *** *p* < 0.001.

Immunohistochemical staining showed that considerable F4/80+ cells (macrophages marker) infiltrated, and some CD3+ T cells (pan T cells marker) were observed in vehicle (iv, scj) groups in corneal allografts (Figure 3B,C) when compared with the infiltration of CD3+ T cells. F4/80+ macrophages were reduced in MDSC (iv, scj) injected groups (Figure 3D,E). F4/80+ macrophages were abundantly recruited to the corneal stroma after corneal transplantation. The MDSCs groups showed a significant decrease compared with the vehicle groups (PBSiv vs. MDSCscj *p* < 0.01, PBSiv vs. MDSCiv *p* < 0.001, PBSscj vs. MDSCscj *p* < 0.01, PBSscj vs. MDSCiv *p* < 0.001, Figure 3G). Moreover, the infiltration of CD3+ T cells was significantly suppressed by MDSCs administration (MDSCiv vs. PBSscj *p* < 0.01, MDSCiv vs. PBSiv *p* < 0.001, MDSCscj vs. PBSscj *p* < 0.01, MDSCscj vs. PBSiv *p* < 0.001, Figure 3F). Normal corneas were not observed with infiltrating macrophages and T cells (Figure 3A,F,G).

### 3.4. MDSCs Prevented Cellular Apoptosis in Grafted Corneas

**Figure 4:**
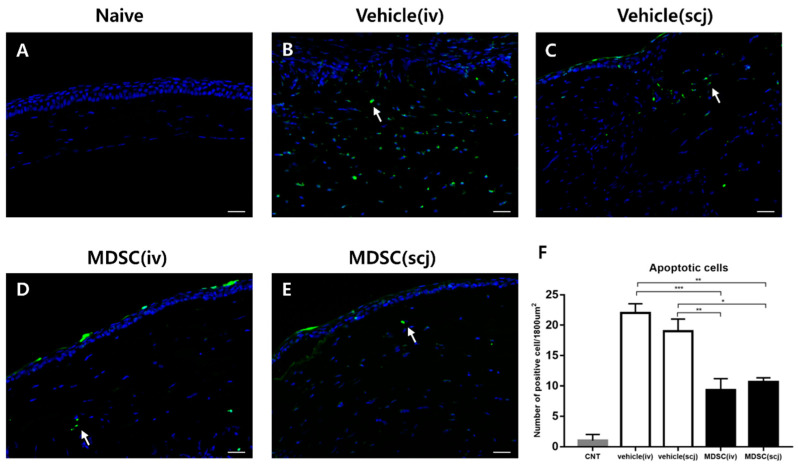
Assessment of cellular apoptosis in the corneal graft 6 weeks after the operation. Representative terminal deoxynucleotidyl transferase dUTP nick end labeling (TUNEL; green; white arrow) assay images (20× magnification, scale bar = 50 µm) of the corneal graft of each group; non-transplanted naive cornea (**A**), PBSiv (**B**), PBSscj (**C**), MDSCiv (**D**), and MDSCscj (**E**). (**F**) Quantitative analysis graph of TUNEL-positive apoptotic cells (green). MDSC-treated groups inhibited the cell death of the grafted corneas compared with that of the PBS-treated group (* *p* < 0.05, ** *p* < 0.01, *** *p* < 0.001). Each experiment consisted of three corneas per group.

F4/80+ macrophage participates in the inflammatory environment and angiogenesis-related to graft rejection. Allogeneic CD3+ T cells are one of the major factors inducing apoptosis in transplanted corneas. Therefore, TUNEL staining was used to confirm apoptosis by immune cells post-transplantation. Representative fluorescence micrographs were taken at the graft and showed that TUNEL-positive apoptotic cells were readily observed; significant differences were also found between MDSCs and PBS groups (Figure 4A–E). MDSCs (iv, scj) groups had significantly inhibited apoptosis compared with the PBS groups (PBSiv vs. MDSCscj *p* < 0.01, PBSiv vs. MDSCiv *p* < 0.001, PBSscj vs. MDSCscj *p* < 0.05, PBSscj vs. MDSCiv *p* < 0.01, Figure 4F). No significant differences were detected between the local and systemic groups of each therapeutic agent in the TUNEL assay (Figure 4F). These results indicated that MDSCs could significantly inhibit immune cell infiltration and graft tissue damage.

### 3.5. MDSCs Alleviated Neovascularization and Lymphangiogenesis on Grafted Corneas

**Figure 5:**
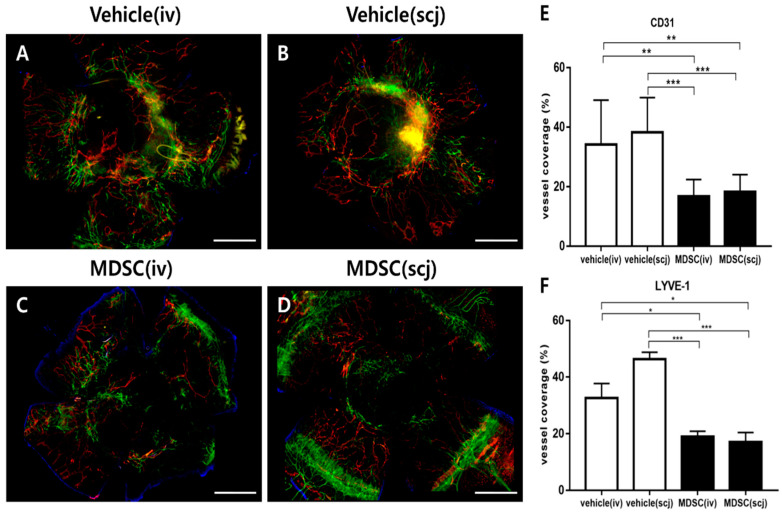
Comparisons of neovascularization and lymphangiogenesis in corneal allografts via MDSC administration. Representative whole-mount corneal immunofluorescent CD31^hi^ (green) and LYVE-1^hi^ (red) staining images from each group: PBSiv (**A**), MDSCiv (**C**), PBSscj (**B**), and MDSCscj (**D**). The representative photograph is a combination of multiple stitched photographs (5× magnification, scale bar = 500 μm) taken after dividing the whole cornea into several parts. MDSC groups (MDSCiv, MDSCscj) presented the significantly suppressed areas of neovascularization (**E**), green-CD31^hi^; white arrows for blood vessels and lymphangiogenesis ((**F**), red-LYVE-1^hi^) rather than those of the vehicle groups (PBSiv, PBSscj). Data are presented as the mean ± SEM of three repeated experiments involving three corneas per group (**E**,**F**); * *p* < 0.05, ** *p* < 0.01, and *** *p* < 0.001.

**Figure 6:**
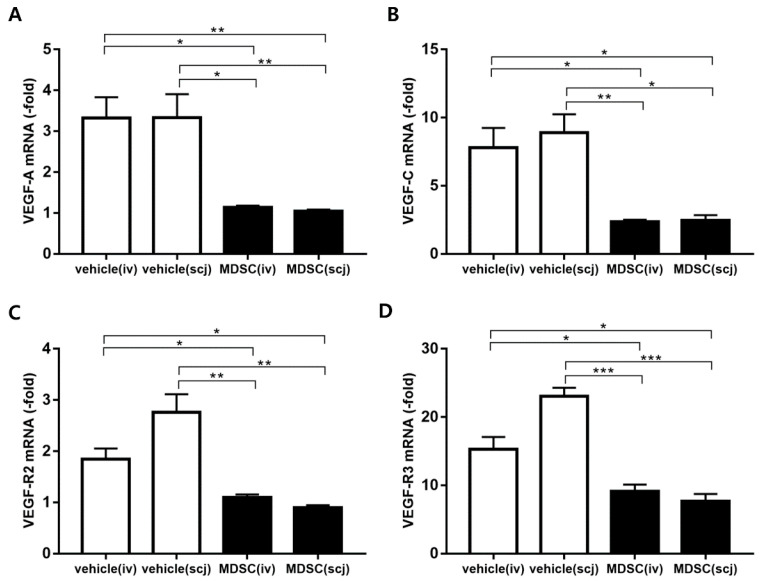
Real-time polymerase chain reaction analysis of the mRNA expression levels of angiogenesis and lymphangiogenesis on the grafted cornea. mRNA levels of angiogenesis ((**A**): VEGF-A and (**C**): VEGFR-1) and lymphangiogenesis ((**B**): VEGF-C, (**D**): VEGFR-3) on the grafted corneas 6 weeks after corneal transplantation. The MDSCs groups (MDSCiv, MDSCscj) were compared with PBSiv and PBSscj (*n* =  3–4, * *p* < 0.05, ** *p* < 0.01 and *** *p* < 0.001). The local and systemic administration of MDSC groups (MDSCiv, MDSCscj) showed a decreased mRNA expression of VEGF-A, VEGFR-1, VEGF-C, and VEGFR-3, in comparison with that of the PBS-treated groups (PBSiv, PBSscj). Data were normalized to GAPDH as internal control, and relative values were expressed as the fold change of the naïve corneas. Data are presented as mean ± SEM of three or four experiments. Each experiment consisted of three or four corneas per group.

The angiogenesis and lymphangiogenesis of the grafts were assessed by fluorescent immunohistochemical staining of platelet endothelial cell adhesion molecule (CD31, known as PECAM-1) and lymphatic endothelial hyaluronan receptor-1 (LYVE-1) at postoperative 6 weeks. The representative photographs of the immunofluorescence-stained neovascularization and lymphangiogenesis were shown on whole grafted corneas of each group (Figure 5A–D). The corneal whole-mount staining was analyzed as a graph by quantifying the areas of blood vessels and lymphatic vessels in each total corneal area (Figure 5E,F). MDSCs (scj or iv) groups had a significantly decreased CD31^hi^ neovascularization area in the quantitative graph of the whole mount corneas (PBSiv vs. MDSCiv *p* < 0.01, PBSscj vs. MDSCscj *p* < 0.001, Figure 5E). The quantification of the lymphangiogenic area revealed that the systemic or local injections of MDSCs clearly reduced LYVE-1^hi^ lymphangiogenesis in the whole corneal mounts (PBSiv vs. MDSCiv, *p*  <  0.05; PBSscj vs. MDSCscj, *p* < 0.001, Figure 5F). Furthermore, the mRNA expression levels of angiogenesis (VEGF-A, VEGFR-1) and lymphangiogenesis (VEGF-C, VEGFR-3)-related genes were assessed in extracted grafted corneas at 6 weeks post-transplantation (Figure 6). The mRNA expression of VEGF-A, VEGFR-1, VEGF-C, and VEGFR-3 significantly decreased in the MDSCiv group, compared with the PBSiv group at 6 weeks (*p* < 0.05, *p* < 0.05, *p* < 0.05, and *p* < 0.05, respectively, Figure 6A–D). The local delivery of MDSCs significantly suppressed the levels of angiogenesis- and lymphangiogenesis-related gene expression compared with those in the PBSscj group (*p* < 0.01, *p* < 0.05, *p* < 0.01, and *p* < 0.0001, respectively, Figure 6A–D). No significant difference in CD31^hi^ and LYVE-1^hi^ areas (%) occurred in the whole corneal staining between MDSCs groups, even at a lower dose in the MDSCscj group than in the MDSCiv group (Figure 5E,F). In addition, no significant differences in the levels of angiogenesis- and lymphangiogenesis-related mRNA genes between MDSCs groups were noted (Figure 6).

## 4. Discussion

The immune privilege of a healthy cornea, characterized by immunomodulatory factors in the eye and the absence of vascularization and lymphangiogenesis, is an important factor in preserving corneal transparency and graft survival in corneal allograft models [28,29]. The breakdown of immune privilege through inflammation, trauma, surgery, and infections, can induce the growth of blood vessels and lymphatics into the cornea and increase the risk of graft rejection by increased exposure of alloantigens to the immune system [7,30,31,32]. In our study, the local and systemic administration of CB-MDSCs suppressed graft rejection in the corneal allograft and positively affected graft survival. However, studies have yet to investigate ocular MDSC injection, which has clinical advantages in corneal allografts over systemic injection. Our data presented that the local/subconjunctival injection of relatively lower doses of CB-MDSCs rather than the systemic injection prolonged corneal graft survival and contributed to potential immune modulation on graft rejection. Umbilical cord blood (UCB) is a source of MDSCs, because UCB DCs express lower levels of MHC class II, CD80, and CD86, compared to the peripheral blood, and they are rich in MDSCs, which could have a prominent immunomodulatory potential and reduce the risk of HLA mismatched related immunologic responses among others adult sources. [33] Therefore, UCB-derived MDSCs could be a good candidate for further promising clinical trials.

We confirmed that the ocular administration of MDSCs on days 0 and 7 significantly prolonged graft survival in an allogeneic corneal transplant model for up to 6 weeks. Previous studies showed that the intravenous adoptive transfer of MDSCs delays graft rejection in a corneal allotransplantation model by regulating T-cell activation [34]. Indirect allorecognition and T cell activation are the main immunopathogeneses of corneal allograft rejection. Alloantigens from the grafted donor cornea is processed by MHC class II APCs, which present processed antigens to the recipient’s naive T cells, generating alloreactive T cells in draining LNs (10, 15, 16). In the present study, we identified the regulation of IFN-γ+ CD4+ cells and MHC class II^hi^ CD11c+ APCs that play an important role in corneal allograft rejection. Previous studies demonstrated that human MDSCs secrete inhibitory mediators (such as IL-10 and TGF), inhibit TLR ligand-induced IL-12 production of DCs, and impair T cell stimulatory functions of DCs in animal models [34,35,36]. Dietz S et al. demonstrated that MDSCs are capable of downregulating HLA class I/II molecules and upregulating co-inhibitory molecules, including programmed death ligand-1/2 [37]. In our study, the reduced infiltration of CD11c+ MHC II^hi^ cells in draining LNs of the MDSC groups indicated that CB-MDSCs could suppress the maturation and migration of DCs. Interestingly, the subconjunctival injection of lower-dose MDSCs presented a higher inhibitory activity of DCs maturation than systemic injection. 

The infiltrations of IFN-γ secreting CD4+ and CD8+ T cells into graft sites can aggravate the inflammatory environment and expand the recruitment of more T cells and F4/80+ macrophages into grafted corneas [26,32]. In a healthy cornea, the immune privilege of the eye suppresses antigen-specific delayed-type hypersensitivity [38] mediated by anterior chamber-associated immune deviation [38,39]. However, impaired immune privilege after corneal transplantation leads to antigen-specific delayed-type hypersensitivity, which induces donor antigen-specific T cell proliferation and infiltration of macrophages into graft sites [39]. Macrophages are also a critical factor in corneal graft rejection as they further recruit immune cells and aggravate inflammatory responses by locally producing IFN-γ and tumor necrosis factor. Our data presented that CB-MDSCs administration suppressed the infiltration of CD3+ T cells and F4/80+ macrophages in allograft corneas. In addition, the maturation of CD11c+ DCs and the expansion of IFN-γ+ CD4+ T cells in draining LNs were significantly decreased by MDSCs injection. Previous studies reported that MDSCs can produce anti-inflammatory mediators and inhibit CD4+ T cell proliferation mediated by the iNOS expression of MDSCs in animal models [22,35,40]. Jensen, K. P. et al. demonstrated a significant increase of MDSCs early posttransplant in mouse cardiac transplantation and human kidney transplantation humans, which suppressed the proliferation and production of IL-2 and IFN-γ on T cells [41]. Inflammatory mediators, such as IFN-γ, secreted by CD4+ T cells and macrophages during graft rejection destroy tissues, as shown by a significant decrease in apoptosis at the graft site injected with CB-MDSCs in our study. This finding indicated that the recruitment of allogeneic T cells and macrophages into the grafted corneas was suppressed by MDSCs treatments; therefore, graft rejection-related inflammatory environment and grafted tissue destruction could be alleviated by injected MDSCs.

Allograft rejection creates an inflammatory environment and increases the infiltration of immune cells, such as T cells and macrophages, into grafted corneas. Macrophages produce inflammatory cytokines and VEGFs, which are major causes of angiogenesis and lymphangiogenesis at graft sites [26,42]. Newly developed angiogenesis and lymphangiogenesis are the main causes of corneal immune privilege disruption after corneal transplantation because donor antigen-presenting mature DCs (MHC II^hi^ CD11c+ cells) migrate from the grafted cornea to LNs through lymphatic vessels (efferent loop) and alloantigen-specific T cells in LNs are recruited to cornea via blood vessels (afferent loop) [26]. Thus, corneal angiogenesis and lymphangiogenesis increase after corneal transplantation and promote the migration of mature DCs in draining LNs and the recruitment of CD3+ T cells into grafted sites. Previous studies demonstrated that MDSCs could indirectly suppress angiogenesis and lymphangiogenesis by decreasing inflammation in an iNOS-dependent manner and inhibiting macrophage infiltration and activation in grafted sites [43,44,45,46]. Fujimoto K et al. demonstrated monocytic MDSCs injection prolonged graft survival after murine cardiac transplantation by inhibiting naïve T cell activation in iNOS dependent manner [47]. Cao P et al. presented an adoptive transfer of bone marrow-derived MDSCs significantly prolonged the survival of allo-skin graft and improved immune tolerance through the Arg-1 pathway in the transplant mice. Furthermore, MDSCs could promote the prominent expansion of Tregs to induce an efficient immune tolerance [48].

In our study, the local and systemic injection of CB-MDSCs inhibited angiogenesis and lymphangiogenesis on the whole mount immunofluorescent staining of the corneas and significantly led to the decreased mRNA expression of angiogenesis and lymphangiogenesis-related genes, such as VEGF-A, VEGFR-1, VEGF-C, and VEGFR-3 in the grafted site. Therefore, MDSCs could suppress allograft rejection by inhibiting the maturation and migration of DCs, infiltration of macrophages, and ingrowth of angiogenic and lymphatic vessels in grafted sites.

Among the administration methods of cellular therapeutic agents, the systemic injection has some limitations in clinical applications, and the efficacy of cell therapy mostly depends on the delivery method. Circulating injected cells are largely trapped within various nontarget organs, such as the lung, liver, and kidney, and they pose risks of serious side effects, such as pulmonary embolism and strokes [49,50,51]. The mechanisms of the accumulation of MDSCs under pathological conditions are still unclear [52]. Nevertheless, these problems of systemic injection can be overcome by using local administration in clinical trials because MDSCs can be directly delivered to the diseased site via local administration without being largely trapped in other organs. In addition, local delivery often yields better efficiency than systemic injection under more controlled allocation [53,54,55,56]. In our study, although the processing dose was lower, it was confirmed that the local delivery of CB-MDSCs suppressed allogenic graft rejection with impaired expression of neovascularization and lymphangiogenesis compared with the systemic injection, but the difference was not significant. Therefore, our data suggested that the low-dose local subconjunctival injection of MDSCs was methodologically more stable and efficient than the high-dose systemic intravenous injection. One of the objectives of this study was to confirm the therapeutic efficacy of the local and systemic administration of MDSCs in this model, even at a lower dose of local delivery. However, further studies on variable doses regarding administration routes of MDSCs in this corneal transplantation model will clarify the amount of MDSCs in future clinical trials. Furthermore, there are still many debated issues to be clarified in further investigation. MDSCs could inhibit dysregulated immune responses, such as autoimmune diseases and transplantation, but they also suppress critical immune responses in bacterial and viral infections and tumor growth/metastasis. Therefore, MDSCs could affect physiological and pathological conditions, depending on their environment, such as chronic inflammation and stress. [57] The other concern may be the possibility of the differentiation of immature MDSCs into other immune cells, including DCs, macrophages, or neutrophils, affected by several external environmental conditions [58,59]. In our study, local and systemic MDSCs treatment did not show an increased population of DCs and macrophages at the transplanted cornea, compared to PBS control groups, but we need to evaluate the specific distribution and differentiation of MDSCs systemically in further experiments. Finally, therapeutic cell transfer always holds potential risks, including recruitment of injected cells to nontargeted organs and immune reaction against transferred cells, which need to be studied carefully before the clinical application [58,60].

In summary, our findings suggested that the local and systemic injection of CB-MDSCs could suppress allogenic T cell-mediated graft rejection by inhibiting the maturation of DCs and the pathological development of angiogenesis/lymphangiogenesis at graft sites, thereby suppressing the infiltration of alloantigen-specific T cells and macrophages into the grafted corneas and the migration of mature activated DCs into draining LNs. Interestingly, lower doses of locally administered CB-MDSCs showed similar efficacy in terms of graft survival to that of systemic administration. To our knowledge, this is the first study to compare the efficacy of local and systemic delivery of CB-MDSCs to suppress graft rejection on murine allogeneic corneal transplantation. Therefore, the local delivery of CB-MDSCs would be helpful for corneal graft survival in clinical trials.

## Data Availability

The datasets are available from the corresponding authors upon reasonable request.
